# State-Level COVID-19 Symptom Searches and Case Data: Quantitative Analysis of Political Affiliation as a Predictor for Lag Time Using Google Trends and Centers for Disease Control and Prevention Data

**DOI:** 10.2196/40825

**Published:** 2022-12-23

**Authors:** Alex Turvy

**Affiliations:** 1 City, Culture, and Community Department of Sociology Tulane University New Orleans, LA United States

**Keywords:** COVID-19, search trends, prediction, case, political, symptom, pandemic, data, google, disease, prevention, model

## Abstract

**Background:**

Across each state, the emergence of the COVID-19 pandemic in the United States was marked by policies and rhetoric that often corresponded to the political party in power. These diverging responses have sparked broad ongoing discussion about how the political leadership of a state may affect not only the COVID-19 case numbers in a given state but also the subjective individual experience of the pandemic.

**Objective:**

This study leverages state-level data from Google Search Trends and Centers for Disease Control and Prevention (CDC) daily case data to investigate the temporal relationship between increases in relative search volume for COVID-19 symptoms and corresponding increases in case data. I aimed to identify whether there are state-level differences in patterns of lag time across each of the 4 spikes in the data (RQ1) and whether the political climate in a given state is associated with these differences (RQ2).

**Methods:**

Using publicly available data from Google Trends and the CDC, linear mixed modeling was utilized to account for random state-level intercepts. Lag time was operationalized as number of days between a peak (a sustained increase before a sustained decline) in symptom search data and a corresponding spike in case data and was calculated manually for each of the 4 spikes in individual states. Google offers a data set that tracks the relative search incidence of more than 400 potential COVID-19 symptoms, which is normalized on a 0-100 scale. I used the CDC’s definition of the 11 most common COVID-19 symptoms and created a single construct variable that operationalizes symptom searches. To measure political climate, I considered the proportion of 2020 Trump popular votes in a state as well as a dummy variable for the political party that controls the governorship and a continuous variable measuring proportional party control of federal Congressional representatives.

**Results:**

The strongest overall fit was for a linear mixed model that included proportion of 2020 Trump votes as the predictive variable of interest and included controls for mean daily cases and deaths as well as population. Additional political climate variables were discarded for lack of model fit. Findings indicated evidence that there are statistically significant differences in lag time by state but that no individual variable measuring political climate was a statistically significant predictor of these differences.

**Conclusions:**

Given that there will likely be future pandemics within this political climate, it is important to understand how political leadership affects perceptions of and corresponding responses to public health crises. Although this study did not fully model this relationship, I believe that future research can build on the state-level differences that I identified by approaching the analysis with a different theoretical model, method for calculating lag time, or level of geographic modeling.

## Introduction

### Background

Incidence of COVID-19 cases in the United States has widely varied across states and across time, as have state-level policies and some of the rhetoric heard in response. There has been ongoing investigation about how mitigation measure mandates such as mask wearing [1,2], social distancing [3], and vaccines [4] affect uptake of these measures as well as how they are associated with actual case numbers. One existing study focused on the political dimensions of mandates and cases [5] by trying to understand the broader social forces that are associated with the response to the pandemic and to mandates, but it is challenging to observe and understand the informal behaviors and tacit unreported beliefs that drive state-level differences in case numbers and response to the pandemic. There has been significant ongoing debate regarding theories for why individual behavioral responses to the pandemic have diverged so significantly over time, and many of these theories have involved analysis of political and administrative messaging. This research enters this conversation by exploring the intersection of political affiliation and perceptions of the pandemic. Increases in search traffic seem to reflect individual concerns about the pandemic as well as information seeking for individuals who are experiencing and observing symptoms. This study aimed to specifically analyze the effects that political affiliation has on this dynamic; there has been plenty of concern about the danger of public health becoming more politicized, and this research intended to add granularity to our understanding.

Both anecdotally and in popular media, there is discussion about how trends in COVID-19–related Google searches might be associated with ongoing COVID-19 case numbers as reported to the Centers for Disease Control and Prevention (CDC) [6]. Using Google Trends search data about COVID-19–related symptoms along with CDC data concerning state-level case numbers, I investigated 2 questions:

1. RQ1: Are there state-level differences in the lag time between spikes in searches for COVID-19 symptoms and spikes in reported COVID-19 cases?
2. RQ2: If these state-level differences do exist, do covariates related to political leadership contribute to state-level variance in lag time?

I hypothesized that state-level political outcomes, as a marker of the dominant or collective political identification of a state’s voters, offer a route to investigating how social behavior via self-identified group affiliation explains differences in the temporal relationship between spikes in COVID-19–related searches and later spikes in total confirmed COVID-19 cases. I expected to find that political variables marking Republican identification are associated with a decrease in lag time, given what we know about the differences in compliance with mitigation measures and vaccine uptake and what this suggests about broader COVID-19 risk beliefs and self-surveillance of symptoms. This lag time relationship may give us insight into how people think about COVID-19: Are they proactive about watching for and managing symptoms, or do they only start noticing symptoms once cases begin to increase and spike?

### Literature Review

#### Theoretical Approach

Social cognitive theory (SCT) [7] frames learning and behavior socially, noting that there is a reciprocal relationship between an individual, their environment, and their behavior—while emphasizing the specifically social nature of this triad. That is, people tend to learn through observing the actions of those in their environment along with their own experiences. The essential lens to understand SCT in this context is how it focuses specifically on personal but environmentally contextualized agency. The social identity approach (SIA) by Abrams and Hogg [8] is a complementary perspective, adding that not only is learning and behavior social but also that knowledge of being in social groups affects how people attach emotion and value to certain behaviors and circumstances. They also emphasize the influence of one’s own in-groups and out-groups as part of this individual/group relationship.

In terms of compliance with health behaviors, the research has settled around 3 major factors that tend to drive an individual’s level of compliance: perceived risk to oneself, belief in behavior effectiveness, and observed risk to others. During the H1N1 influenza pandemic, a review of 26 studies found consistently strong associations between an individual’s perceived susceptibility to the virus and increased compliance with recommended behaviors; this effect was strengthened when perceived severity of infection increased and was consistent across many countries and cultures [9]. Perceived risk to self is affected by factors such as perceived personal vulnerability [10], level of cultural individualism [11], fear [12,13], and anger [14].

Belief in the effectiveness of health behaviors is driven by a number of affective and epistemological factors as well; laypeople tend to create their own justifications for health behaviors by pulling from not only both establishment and nonestablishment sources [15] but also their preferred mass media sources [16]. Although this belief is reduced in all groups when they perceive recommendations to predominantly be moralistic [17], general trust in government is strongly associated with affecting perceived risk in complicated and sometimes counterintuitive ways [10,17,18]. Finally, observed risk to specific others and a more generalized community tends to be positively affected by a general sense of conscientiousness [12]. This is true when individuals feel an ethical responsibility to their community [19] but is also true on a more individual level when individuals experience the vulnerability of their close ties [20] and so avoid the perception that health concerns are “overhyped” [21].

In this paper, I do not explore the details of how particular political ideologies specifically affect compliance behaviors. Instead, the context described in the previous paragraphs serves to highlight the power that political identities and communication have on individual compliance. From both Democratic and Republican politicians, we frequently hear justifications for COVID-19 behaviors that speak directly to these 3 factors. These justifications are often oriented around personal ethical responsibility, the meaning of a sense of community, and the epistemological justifications for agency recommendations; each of these threads is an important part of building the set of beliefs that ultimately drive behaviors. More recent research extends these claims further, demonstrating that political affiliation is associated with particular pandemic responses that cannot be explained solely via these 3 factors [5], highlighting how at least part of an individual’s response is related to affinity-related dogma or that operational constraints and diverging priorities tend to be privileged over more objective risk assessments [22].

#### Using Search Data in Public Health Research

Public health professionals and researchers have broadly been discussing the value of search data for both detection and surveillance for quite some time, initially highlighting its value in a landmark paper that advocated for its use but cautioned that it should be predominantly used in areas with widespread internet access [23]. Given that American internet usage is now frequent and widespread across most settings, researchers have been able to turn their attention specifically to its use in early detection of emergent diseases [24].

There is also evidence that search volume is effective for ongoing monitoring and surveillance, both for active and predictive surveillance [25], and for passive or retrospective surveillance that aims to understand how factors such as the media affect the relationship between search interest and cases [26].

Search data have been used as a lens specifically for understanding COVID-19 data, but this research has had different areas of focus: searches as predictive of local metropolitan-level data [27,28], impacts on mental health [29], and more rare symptoms (anosmia and ageusia) as ineffective predictors of case incidence [30]. Eysenbach [31] took a somewhat similar approach to my own but concerning flu symptoms and incidence instead of COVID-19, finding strong correlations between clicks on sponsored flu-related links and flu diagnoses 7 days later.

There are ongoing challenges to using search data as well as other novel data streams (NDS) such as social media posts; although there is some evidence that they can help to retroactively explore associations and wield predictive power, there are still unresolved issues of how to assess reliability and validity of these data [32]. Some challenges such as lack of transparency and reproducibility [33] can be resolved by establishing accepted best practices such as sharing specific search strings and Boolean operators, but others are more related to complex sociological and psychological phenomena such as a panic-induced search increase that will likely prove to be much more difficult to solve [34]. Given the established value and unresolved challenges of using NDS such as search traffic, the use of Google Trends data should be seen as a supplemental tool for public health researchers along with more traditional and localized practices instead of as a substitute [35].

This paper addressed these methodological limitations by using search data indirectly; although this means that some of these concerns become endogenous to the modeling, it is beneficial insofar as this captures these complex dynamics within the lag time variable. Instead of relying on search trends data as an accurate predictive or surveillance tool, I used it to highlight areas of difference across regions and explore the reasons for those differences.

## Methods

Although there is discussion in popular media about using trends data in a predictive way and some push toward this in technical methods literature [36], there is not yet evidence that search data are defensible for use in a predictive way about future health trends [30] given that access to real-time or otherwise timely raw data is not possible. Instead, trends data are most useful for monitoring and evaluating relationships between events in the past, especially as one predictive element within a larger model [37,38].

For the purposes of this study, I used the publicly available COVID-19 Search Trends data set, which tracks the incidence of more than 400 symptoms associated at various levels with COVID-19. Typically, Google does not allow for large-scale downloads of granular daily search trends data except through use of their API. However, the company made this COVID-19–specific search data available specifically for researchers and journalists; the data include both daily data as well as state-level geographic data. The data set allows for what Google calls “metro areas,” but these do not include shapefiles that could be used to match the search data with CDC data via geographic information system (GIS) software. Like all Google Search Trends data, it is normalized on a 0-100 scale, contextualized within the geography and time range in question, and based on a particular search string’s incidence in proportion to all searches in that same geography and time frame. This study used daily trends data from each state for the time period from March 11, 2020, through April 4, 2022, and was retrieved on April 15, 2022, via Google’s internally hosted GitHub.

The beginning of the study period is the day on which COVID-19 was declared a global pandemic by the World Health Organization, and the end of the period is the final day of trends data from Google’s data set; so, the total number of days in the study period is 762. Although not every region experienced their first case by the beginning of the trends data time period, I was specifically interested in the lag time between increases in searches and increases in cases. Given that there was already widespread discussion of COVID-19 in popular media and so this is broadly reflected in the search data, search volume was already increasing across all regions by March 11, 2020, and this allowed me to examine state-level differences in when cases began to increase.

The CDC makes daily data detailing new cases, new hospitalizations, and new deaths associated with COVID-19 available to the public. These data are available to the county level, but this study exclusively used state-level data in order to look at these variables alongside the state-level search trends data. These data are raw numbers, but population is controlled for in regression modeling to account for this. There are likely data gaps related to submission logistics and other issues; additionally, the data had some level of daily noise within the data as states catch up with missed submissions and correct submissions from previous days, but this has been accounted for in the process of examining the data closely in the calculations for lag time as the dependent variable.

The analytic strategy used a linear mixed model with fixed effects for all included predictors and controls and random intercepts for each state to investigate the state-level differences named in the research questions. Additionally, I included a random effect for the political predictor nested within state clusters, recognizing how SCT indicates that behavior is affected in an ongoing reciprocal way by environment—here, the state and its political climate are considered as that environment.

I called the key outcome variable “lag time,” and it measures the amount of time in days between a spike in COVID-19 symptom searches and a (typically) later corresponding spike in reported COVID-19 cases. This variable was calculated manually using the raw data and plots as a guide. Each state had 4 identifiable case peaks of varying magnitudes. After marking these and accounting for any noise or reporting gaps in the data, I turned to the search data to identify whether there was a corresponding spike in symptom searches that preceded the case spike. In nearly all cases, there was an associated spike, and this was measured in number of days.

Political variables under consideration included the proportion of Trump popular votes within a state in the 2020 election, a dummy variable indicating whether a Republican holds the Governor office, and a variable measuring the proportion of a state’s federal representatives in the House of Representatives and Senate that is Republican. The latter 2 variables did not lead to a strong model fit in any case and were discarded, so the Trump proportion variable remained as the main predictor in this model. Controls for mean daily cases, mean daily deaths, and state population were also included in the model. All predictors and controls were normalized using *z* scores.

Within the search data, I identified the 11 most common symptoms of COVID-19 as reported by the CDC and created a construct to represent the collective incidence of these search terms. These symptoms included headache, nasal congestion, rhinorrhea, fever, sore throat, nausea, anosmia, ageusia, fatigue, and diarrhea. This symptom construct has a Cronbach alpha score of .812. An alpha value greater than .8 generally indicates a strong level of construct reliability. Reliability analysis showed that the alpha value would not be improved by removing any variable from the construct.

Descriptive analysis for daily new case and death data by state, initial bivariate linear regression modeling, calculations for construct reliability, lag time calculations, mixed modeling, and model comparisons were all completed in R. The primary packages used were lubridate for parsing date variables, plotly for examining ggplot2 results in more detail, lme4 and lmerTest for fitting linear mixed models, sjPlot for plotting data to test model assumptions, stargazer for table and figure creation, and all of the packages within the “tidyverse” (primarily dplyr and ggplot2) for cleaning, organizing, and preparing data for analysis and presentation.

## Results

Descriptive statistics for all variables included in the final model are included in Table 1. These data are the raw numbers, but predictors and controls were standardized for analysis to control for the vastly different scales for many of the variables. Given the extremely large volume of daily case and search data, this is not included in this table.

**Figure 1:**
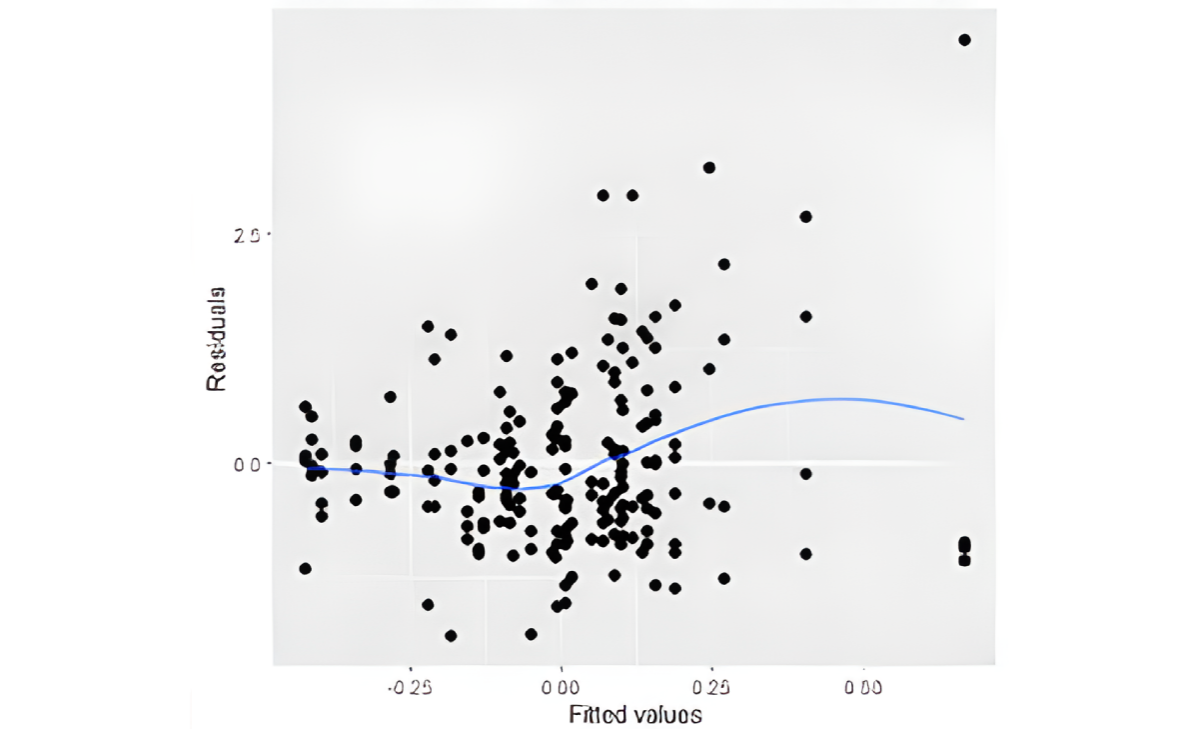
Homoscedasticity (constant variance of residuals). Note: amount and distance of points scattered above and below the line is equal.

Assumptions for linear mixed model regression were checked, confirming that there was a linear relationship between the predictor and outcome variable and that the residuals were independent, uncorrelated, and normally distributed. The residual plot for homoscedasticity of residuals is in Figure 1.

A linear mixed model was fitted using the proportion of 2020 Trump votes within a state as the primary predictive variable. This variable was also nested within state-level clusters to allow its effect to vary within each state. Models using the proposed Governor and Congressional proportion variables were discarded due to comparatively poor model fit statistics. Akaike information criterion (AIC) and Bayesian information criterion (BIC) scores for discarded models were in the range of 600 to 800 points higher, indicating poorer fit.

**Table 1:** Descriptive statistics.

State	Case peaks					Search peaks					Proportion of Trump votes		GOP governor		GOP proportion of Congress		Population		Daily cases, mean		Daily deaths, mean
	1	2	3	4	1		2	3	4												
AL	185	349	584	728	157		342	581	707	0.62		1		0.83		5024803		1700.48		25.48	
AK	187	319	612	729	176		273	589	714	0.53		1		0.83		732441		314.21		1.38	
AZ	162	348	585	732	156		342	579	718	0.49		1		0.45		7177986		2639.60		39.21	
AR	199	346	576	729	156		342	496	718	0.62		1		0.92		3012232		1092.82		14.46	
CA	183	354	589	727	169		344	463	714	0.34		0		0.16		39499738		11713.8		116.06	
CO	189	297	457	717	181		302	461	708	0.42		0		0.39		5784308		1775.10		15.75	
CT	92	317	605	720	52		294	567	707	0.39		0		0.00		3600260		969.89		14.17	
DE	104	354	598	718	57		322	567	708	0.40		0		0.00		991886		341.14		3.76	
FL	172	345	583	718	157		342	567	708	0.51		1		0.58		21569932		7696.68		96.48	
GA	185	353	591	713	158		343	579	707	0.49		1		0.61		10725800		3628.51		48.40	
HI	204	287	586	707	174		273	537	701	0.34		0		0.00		1451911		308.27		1.80	
ID	176	323	631	729	119		300	592	727	0.64		1		0.75		1847772		583.05		6.43	
IL	101	297	444	720	57		293	462	708	0.41		0		0.30		12785245		4045.41		49.53	
IN	97	317	598	720	57		293	579	708	0.57		1		0.82		6785644		2218.97		30.79	
IA	102	297	602	724	92		293	601	714	0.53		1		0.67		3188669		990.51		12.42	
KS	100	307	597	729	89		301	518	714	0.56		0		0.83		2935880		1010.45		11.14	
KY	101	351	590	731	54		344	579	707	0.62		0		0.88		3503958		1532.39		22.52	
LA	173	351	590	713	157		342	554	707	0.58		0		0.82		4651203		1532.39		22.52	
ME	119	352	450	722	107		293	448	707	0.44		0		0.12		1362280		312.94		2.98	
MD	119	318	613	719	111		317	615	707	0.32		1		0.10		6172679		1334.10		18.86	
MA	94	3353	603	720	53		350	567	708	0.32		1		0.05		7022220		2246.86		31.38	
MI	115	294	440	728	111		293	442	708	0.48		0		0.44		10067664		3134.87		39.68	
MN	107	298	441	729	105		300	462	714	0.45		0		0.35		5707165		1869.44		16.37	
MS	181	353	576	728	157		342	578	707	0.58		1		0.83		2956870		1029.39		14.62	
MI	98	293	560	713	55		259	567	708	0.57		1		0.80		6154481		1841.18		22.75	
MT	107	298	643	734	57		257	641	719	0.57		1		0.67		1086193		355.38		4.24	
NE	107	302	603	727	57		301	574	714	0.58		1		0.80		1961455		598.27		4.97	
NV	177	352	598	728	169		343	587	714	0.48		0		0.33		3114071		895.19		13.34	
NH	101	348	444	728	57		259	400	714	0.45		1		0.00		1377848		399.08		3.23	
NJ	73	354	604	717	57		350	567	707	0.41		0		0.07		9279743		2919.76		41.26	
NM	188	303	569	728	174		302	567	707	0.43		0		0.09		3117566		680.66		9.39	
NY	78	353	605	717	57		350	567	707	0.37		0		0.28		21054933		3541.09		36.21	
NC	184	346	590	723	158		342	596	708	0.50		0		0.72		10457177		3452.16		30.48	
ND	121	297	440	729	113		295	449	707	0.65		1		0.67		778962		314.86		2.95	
OH	89	322	598	725	57		302	595	708	0.53		1		0.71		11790587		3502.58		45.09	
OK	168	343	582	727	158		343	550	718	0.65		1		0.93		3962031		1358.26		17.05	
OR	180	318	584	728	128		300	567	718	0.40		0		0.14		4241544		927.88		9.70	
PA	79	325	448	718	57		314	462	708	0.49		0		0.50		12989625		3635.72		58.22	
RI	108	317	602	716	106		258	568	709	0.39		0		0.00		1096229		454.25		4.62	
SC	181	353	584	732	158		342	587	708	0.55		1		0.83		5130829		1927.38		23.20	
SD	109	296	679	729	57		301	576	707	0.62		1		1.00		887099		310.05		3.79	
TN	192	330	599	730	158		343	582	707	0.61		1		0.82		6920119		2633.41		30.82	
TX	178	350	596	722	158		343	587	707	0.52		1		0.66		29217653		8525.78		113.08	
UT	177	398	664	728	126		300	596	714	0.58		1		0.92		3281685		1217.06		6.19	
VT	74	436	604	728	57		460	561	715	0.30		1		0.00		642495		144.45		0.78	
VA	125	361	597	720	111		342	601	708	0.44		1		0.31		8632044		2202.54		25.94	
WA	167	322	561	737	113		301	567	715	0.39		0		0.25		7718785		1920.45		16.46	
WV	183	346	612	713	159		265	553	707	0.69		1		0.80		1789798		653.50		8.81	
WI	143	297	623	727	111		293	615	707	0.49		0		0.60		5892323		2085.48		18.84	
WY	189	301	589	748	159		301	603	722	0.69		1		1.00		577267		204.82		2.35	

The model results are in Table 2; note that the scales displayed for predictors and controls are standardized, but lag time remains measured in days. I used 762 observations for 50 states to calculate 4 intervals of lag time, which led to a sample for the model of 50 states, or units of analysis with 4 repeated measurements per state. The model is a significant improvement over the null model with a single predictor (Trump proportion), no controls, and random intercepts for states. The AIC and BIC scores for the null model were 1776.04 and 1785.93, respectively—the selected model’s scores were 588.85 and 618.535, respectively, and so were a significant improvement in terms of model fit.

However, none of the predictors within the final model were statistically significant, even those that were considered but excluded from the model (Governor party and Congressional delegation parties) because of worse overall model fit. The only significant independent variable in the selected model was mean daily deaths, which had a small negative relationship with lag time, at *P*<.10.

**Figure 2:**
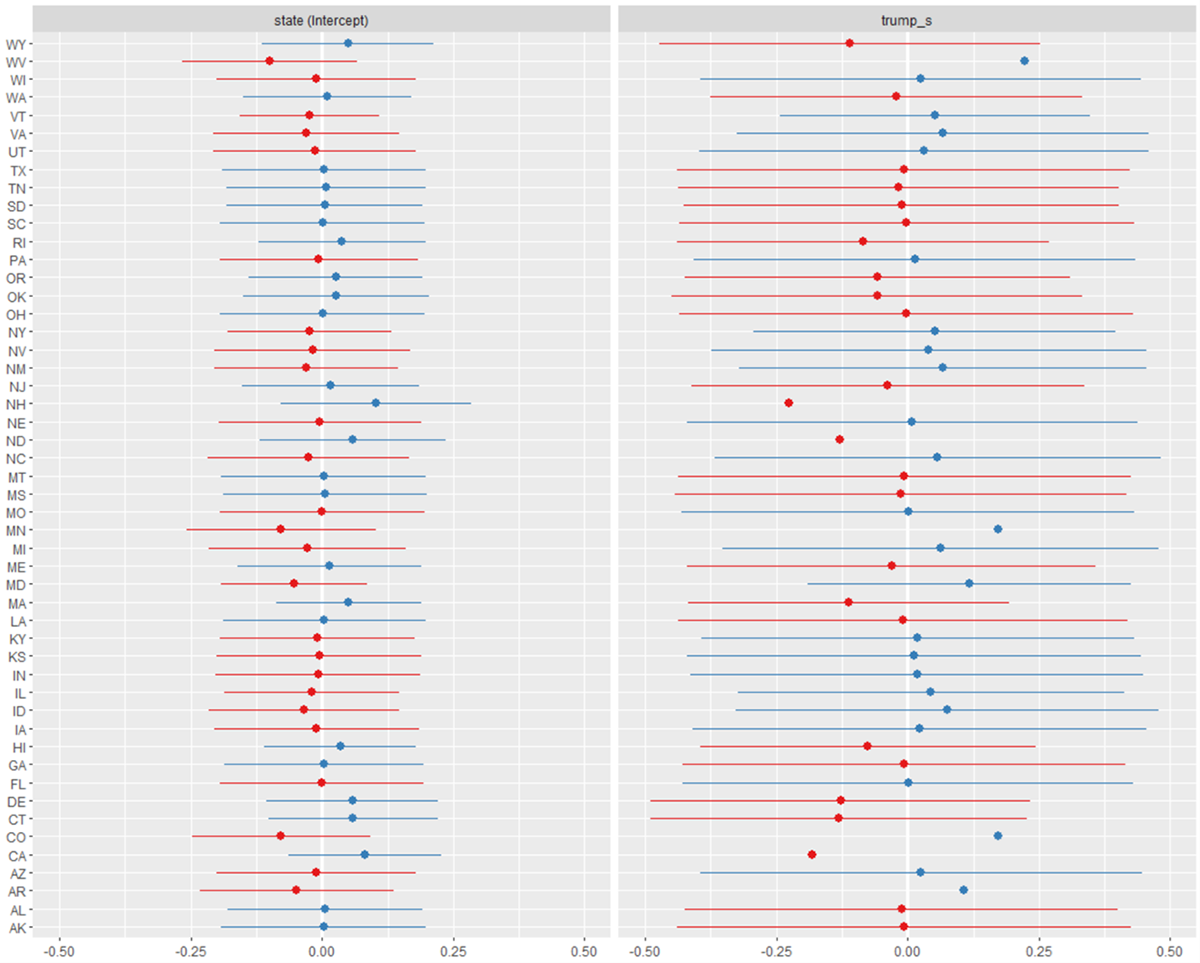
Random effect estimates (intercept and 95% CI) in lag time by state; red: negative; blue: positive.

Random elements in the model were individual state-level intercepts and Trump proportion nested within state as a random effect. There was sufficient variance (σ^2^_state_=0.099 and σ^2^_trump_=0.049) in this random portion of the model to justify their inclusion. The random intercepts for state clusters captured a portion of and thereby reduced the fixed effect residuals, but the model itself lacked predictive power. Thus, the model’s fixed effect error term still captured a relatively high amount of the variance in the data. As Figure 2 shows, there was a range of positive and negative values for each effect, but the relatively wide confidence intervals (95% CI) were another artifact of the relatively low predictive power for this model.

**Table 2:** Linear mixed model results (dependent variable is lag time in days).

Variable		Statistic	*P* value
Proportion of Trump 2020 votes, β (SE)		0.063 (0.091)	.49
Mean daily cases, β (SE)		0.461 (0.569)	.42
Mean daily deaths, β (SE)		–0.670 (0.356)	.07
Population, β (SE)		0.168 (0.430)	.70
Constant, β (SE)		–0.012 (0.076)	.88
**Overall model**			
	Observations	200	N/A^a^
	Log likelihood	–285.425	N/A
	Akaike information criterion	588.850	N/A
.	Bayesian information criterion	618.535	N/A

^a^N/A: not applicable.

## Discussion

### Principal Findings

I have identified evidence to support a positive answer for the first research question but not the second. Results suggest that yes, there are meaningful state-level differences in the lag time between spikes in COVID-19–related search traffic and spikes in COVID-19 cases. However, my hypothesis that political covariates would contribute to a portion of this variation in a statistically significant way was not supported. My findings indicate that including proportion of 2020 Trump voters in the final linear mixed model leads to a model fit that is overall much stronger than a null model with control variables only, but this political affiliation variable is not itself statistically significant or appropriate for predictive inference.

Although this is a partially negative finding, it challenges further work to explore how environmental factors and social group forces (both structural and interpersonal) may cause diverging responses to shared societal crises. The statistically significant differences in lag time across states demonstrate that there are meaningful differences across states that are at least partially causing these changes. I propose 3 potential causes for the mismatch between my theorized model and the results that also suggest directions for future research: level of geographic modeling, approach to calculating lag, and theoretical model mismatch.

### Level of Geographic Modeling

My approach considered state as the geographic unit primarily for logistical reasons. State is an easily available unit in both the trends and cases data, meaning that they can be reliably matched for analysis. However, in doing so, I lost the opportunity for more localized nuance in my political covariates: Even using local city elections as a proxy for political identification might lead to stronger model fit and predictive power, but it may also be possible to use smaller blocks such as census tracts to map the trends data onto already narrowly geotagged CDC case data. However, the Google search data are limited by the lack of GIS shapefiles as well as the lack of city or tract-level data for areas outside of its large metro areas. An analysis with more granular geographic modeling would require the theoretical model to be reconsidered, given that it would exclude data from smaller cities and rural areas. If these localized variables were included in a hypothetical model, it would also likely be wise to include controls such as income and educational attainment, assuming that these are available for the census tracts or areas in question. On a smaller scale, these controls would likely contribute more significantly to a model than the same statistics at a broad state level.

### Approach to Calculating Lag

Although I believe that the method I used to calculate lag is defensible, it is possible that another approach may uncover a significant relationship that is not present here. Pelat et al [39] provided one option within the same domain, describing a method that calculates correlations between increases in searches and incidence of a disease at predefined intervals (eg, 1 week, 1 month). These correlations were stored and used for further regression analysis along with selected predictors.

Effenberger et al [40] took another approach that accounts for lag specifically via time lag correlational analysis. Instead of calculating lag as a repeated measure in longitudinal analysis, they instead mapped multiple models as a network, examining how associations changed at predefined time intervals. This would require a significant reconfiguring of how the research questions were operationalized but may uncover relationships that were not established here.

### Theoretical Model Mismatch

In short, my proposed theoretical model was that, per Bandura’s SCT [7], individuals act as part of a constantly changing and reciprocal triad that is bound by personal factors, environmental influences, and past behavior. As part of this triad, the SIA also tells us that individuals sort themselves into groups by categorizing others, giving meaning to those categories, and then self-sorting into one of these groups. Given how politicized the ongoing cultural response to COVID-19 and mitigation measures has been, I suspected that the competing political understandings of the nature of the pandemic and appropriate reaction would be part of these social processes. Eventually, I hypothesized that this dynamic would affect how people managed their own symptom surveillance and perceived risk, meaning that we would discover differences in lag time between search incidence and case incidence. This theoretical model was not supported by the data here.

It is possible that another theoretical model, operationalized with a different set of predictors, would generate a significant statistical model to explain lag time variation. Barber and Pope [41] approached political identity by investigating how one’s political party identification is associated with his or her individual political ideology; one element from their model that is missing here is the influence of fellow members of a political group. They also define the concepts of “party loyalist” and “policy loyalist” in the context of Trump’s election, which represented an opportune time for investigating this relationship, as ideology and party often diverged. It may be possible to capture these concepts in a more localized model as described in the previous paragraphs.

Another potential theoretical model comes partially from Agadjanian and Lacy [42], who investigated how individual political leaders have a more significant influence on public opinion and behavior than party or ideology. This suggests that, if we could capture political leader characteristics and ideology with some level of granularity, this could be folded into the proposed SCT/SIA framework. As an example, coding leader rhetoric (whether manually or via sentiment analysis) would be possible under both a localized (city or tract-specific) or state-level model.

Notably, a limitation of my approach to geographic modeling is that it does not allow for investigation into whether state-level data are reliably correlated with local provincial data; future research could address this limitation via a study that specifically considers larger cities in comparison with their states or even extend this via propensity score matching at the metro and state levels. Further, the way that I chose to operationalize political identity and affiliation is suboptimal because of the existing geographic constraint. In taking an approach that allows for more geographic granularity, future studies could also model for more granular political variables such as voting by census tract, city council representatives, and mayors.

Although there may be structural, demographic, and geographic factors that contribute to these differences, I believe that the effects of political affiliation and its rippling effects are also closely tied to these significant differences in lag time across states. In the context of not only increasing political polarization and opportunities for political speech but also changing laws and norms around American federalism that may give states more control over shared social functions, it is more important than ever to understand how political identity and political communication affect the physical well-being of a state’s residents.

## Abbreviations

AIC: Akaike information criterion
BIC: Bayesian information criterion
CDC: Centers for Disease Control and Prevention
GIS: geographic information system
NDS: novel data streams
SCT: social cognitive theory
SIA: social identity approach
